# Repetitive Transcranial Magnetic Stimulation to Maintain Treatment Response to Electroconvulsive Therapy in Depression: A Case Series

**DOI:** 10.3389/fpsyt.2013.00073

**Published:** 2013-07-23

**Authors:** Yoshihiro Noda, Zafiris J. Daskalakis, Cinthia Ramos, Daniel M. Blumberger

**Affiliations:** ^1^Department of Psychiatry, Faculty of Medicine, University of Toronto, Toronto, ON, Canada; ^2^Temerty Centre for Therapeutic Brain Intervention, Centre for Addiction and Mental Health, Toronto, ON, Canada; ^3^Centre for Addiction and Mental Health, Campbell Family Mental Health Research Institute, Toronto, ON, Canada; ^4^Geriatric Psychiatry Division, Centre for Addiction and Mental Health, Toronto, ON, Canada

**Keywords:** bilateral rTMS, ECT, relapse prevention, major depression, maintenance rTMS

## Abstract

Electroconvulsive therapy (ECT) is the most effective treatment for a refractory major depression in the context of both unipolar and bipolar affective disorders. However, the relapse rate within the first 6 months after a successful course of ECT to treat a depressive episode can be as high 50%. Evidence-based strategies to prevent relapse have partial efficacy and are associated with problematic adverse effects limiting their use as long-term treatments. Repetitive transcranial magnetic stimulation (rTMS) has demonstrated efficacy in treatment-resistant depression with a favorable adverse effect profile. Herein, we describe six patients, four with unipolar and two with bipolar depression, where rTMS was used to maintain response after a successful course of acute and continuation ECT. rTMS was administered once or twice weekly, at 120% of the resting motor threshold. Patients received sequential bilateral rTMS (low frequency right: 600 pulses, then high frequency left: 3000 pulses). The site of stimulation was 6 cm anterior and 1 cm lateral from the site of maximum stimulation of the abductor pollicis brevis muscle. Depressive symptoms were monitored with the quick inventory of depressive symptoms-self rated. Five of the six patients were able to maintain their response status from 6 to 13 months at the time of last observation. The use of rTMS may be an important relapse prevention strategy following an acute course of ECT. Controlled studies comparing rTMS to current evidence-based relapse prevention strategies are warranted.

## Background

Electroconvulsive therapy (ECT) is an established and important treatment for severe or treatment-resistant depression (TRD) (1). Despite the superior efficacy of ECT in TRD, relapse rates are high after a successful course of treatment (2–4). With antidepressant monotherapy, patients can have a relapse rate as high as 84% within 6 months after an acute course of ECT (2). Other studies have shown that patients who receive pharmacotherapy in a treatment as usual fashion following ECT relapse at a rate of 51% within 6 months of remission (3). High relapse rates after an acute course of ECT is a major problem for the field of convulsive therapy (4–6).

The best evidence for preventing relapse in depression after an acute course of ECT consists of continuation pharmacotherapy (C-Pharm) or continuation electroconvulsive therapy (C-ECT) (7, 8). A Consortium for Research in ECT (CORE) study found that both C-Pharm and C-ECT are superior to a placebo control in reducing the rate of relapse, however both groups still had relatively high relapse rates of 31.6 and 37.1% over 6 months, respectively (7). The relapse rates at 6 months after an acute ECT course did not differ statistically between the two groups (7). In this regard, C-ECT and C-Pharm are the two evidence-based strategies for preventing relapse after an acute ECT course (9). For C-Pharm the seminal studies used the combination of nortriptyline and lithium (2, 7). The tolerability and narrow therapeutic index of lithium can limit the acceptance of the treatment by patients (10). Furthermore, a recent study demonstrated that both nortriptyline and venlafaxine combined with lithium only reduced the relapse rate to 50% within the first 6 months after remission with ECT (11). C-ECT necessitates ongoing ECT that although at a reduced frequency over 6 months that can delay and prolong recovery from the cognitive adverse effects of the acute ECT course (12). Thus, other treatments that can effectively maintain response with fewer adverse effects are greatly needed.

Repetitive transcranial magnetic stimulation (rTMS) has emerged as a new treatment for TRD (13). Unlike ECT, rTMS does not require anesthesia. rTMS is a non-invasive and well-tolerated treatment for depression that has a favorable adverse effect profile (14). Meta-analyses have reported that the effect size of rTMS treatment for depression is around 0.55 (15, 16) which is comparable to at least a subset of antidepressant medications that have effect sizes in the range of 0.17–0.46 (17, 18). The antidepressant effect of rTMS appears to be as durable as with other antidepressant treatments (19). Early studies suggested that rTMS had almost equivalent antidepressant effect as ECT in non-psychotic major depression (20) with similar long-term outcomes at 3 and 6 months (21). However, the accumulation of data has demonstrated that rTMS does not have as robust efficacy as ECT (16, 22, 23).

Though its efficacy may not be as robust as ECT, rTMS may have a similar mechanisms of action (24, 25), as it induces a micro-electrical current in the underlying cortical tissue and results in localized neuronal depolarization (26, 27). rTMS has the significant advantage of being able to electrically stimulate cortical tissue without the need for sedation. In addition, rTMS has a superior tolerability profile and does not adversely effect cognition (13). Though there are relatively few studies that have examined maintenance of response after an acute course of rTMS, emerging data suggests that a schedule of clustered treatments can maintain response in those that have responded to an acute course of rTMS (28).

Herein we describe the use of rTMS to maintain response in six patients who initially responded to an acute and continuation course of ECT and requested to discontinue the course of ECT due to poor tolerability or cognitive side effects.

## Case Series

### Subjects, clinical measure, and medication

Six patients with TRD were switched to twice or once weekly rTMS after ECT treatment. All the patients had recurrent episodes and had failed at least two antidepressant medications in the current episode. Table 1 shows a descriptive data on the demographic, clinical characteristics of the six subjects. Patients were switched from ECT to rTMS treatment for clinical reasons including: (i) patients did not wish to continue with ECT due to concerns around side effects and the overall tolerability of the procedure, or (ii) patients complained of significant cognitive impairment, or (iii) patients declined the addition of lithium to their antidepressant treatment. Depressive symptoms were assessed using the Quick Inventory of Depressive Symptoms-Self Rated (QIDS-SR) (29) with weekly administration throughout both ECT and rTMS treatment courses. The definition of symptom severity of using the QIDS-SR was: normal (0–5), mild (6–10), moderate (11–15), severe (16–20), and very severe (over 21). Remission was defined by QIDS-SR score less than 6. Response was defined as a 50% improvement from baseline and “partial response” was defined as 25% improvement from baseline. Four women and two men between the ages of 27 and 58 (mean ± S.D = 48 ± 11) are described in this series. Four had a diagnosis of unipolar depression and the remaining two bipolar depression, with refractory type of depression. They were prescribed one or two antidepressant medications during ECT and rTMS treatments. The average *Imipramine*-equivalent dose of the antidepressant medications during the maintenance rTMS period was 294 ± 161 mg/day in these patients (30). Medications were kept constant during the rTMS treatment course with minor dosage adjustments and no absolute changes to antidepressant or augmentation agents.

**Table 1:** **Clinical and demographic characteristics**.

**CHARACTERISTICS**	
Number of patients	6
Age	48 ± 11 (mean ± S.D)
Gender (male/female)	2/4
Diagnosis: bipolar depression	2
Unipolar depression	4
Antidepressant medications** (imipramine-equivalent dose)	294 ± 161 (mg/day)
Number of acute ECT	14 ± 5
Number of continuation ECT	15 ± 9
Number of maintenance rTMS	54 ± 11
Baseline score of QIDS-SR (start of ECT)	17 ± 3
Midterm score of QIDS-SR (start of rTMS)	8 ± 6
Ongoing score of QIDS-SR (last rTMS)	8 ± 4

**During the maintenance rTMS period.

### ECT course

Electroconvulsive therapy was administered using the seizure titration method using a MECTA Spectrum 5000Q. All patients started with right unilateral ultra-brief (RUL-UB) pulse width (0.3 ms) ECT with seizure threshold titration. Subsequent treatment was administered at six times the seizure threshold. This technique has been shown to be an effective treatment with a reduced cognitive side effect profile (31). Two patients were switched to bilateral standard pulse width ECT due to lack of response to RUL-UB and seizure threshold was re-titrated. Bilateral ECT was administered using a pulse width of 1.0 ms at 1.5 times the seizure threshold. The length of acute ECT courses ranged from 9 to 20 treatments twice or three times weekly. The average acute course length was 14 ± 5 (mean ± S.D) treatments. All but one went onto a course of C-ECT course that ranged from 8 to 31 treatments prior to switching to rTMS. The average length was 15 ± 9 (mean ± S.D) continuation ECT treatments.

### rTMS course

Repetitive transcranial magnetic stimulation was administered using a MagPro RX100 Repetitive Magnetic Stimulator (Magventure, Denmark) and a hand-held B65, figure-of-8 coil. rTMS was administered once or twice weekly, at an intensity of 120% of the resting motor threshold. The intensity was administered at as a percentage of the maximum stimulator output (MSO). For example, if the resting motor threshold was 40% MSO the intensity of treatment would be delivered at 48% MSO. Choice of one or two treatments per week was based on the severity of depression and patient’s compliance to the treatment. Patients received sequential bilateral rTMS using low frequency right (LFR) for 600 pulses in one continuous train, then high frequency left (HFL) for 3000 pulses. For the HFL stimulation the number of pulses per train was 50, for 60 trains with an inter-train interval of 20 s. The site of stimulation was 6 cm anterior and 1 cm lateral from the site of maximum stimulation of the abductor pollicis brevis muscle. This location has been advocated as more accurately targeting the dorsolateral prefrontal cortex (DLPFC) (32, 33). The rTMS protocol was based on studies showing demonstrating efficacy with sequential bilateral rTMS (34, 35). The frequency was chosen based on clinical experience in our center with maintenance rTMS. One patient was switched from sequential bilateral rTMS to an extended, 6000 pulse, session of HFL rTMS alone (120 trains of 50 pulses with an inter-train interval of 20 s). The length of the maintenance rTMS course, at last observation, ranged from 44 to 66 treatments (mean ± S.D = 54 ± 11) which translates to a mean of 9 months. Patients were offered rTMS on compassionate grounds based on their history of difficult to treat depression. Prior to consenting to rTMS, patients were informed of the rationale for using rTMS to maintain response and the limitation that using rTMS in this context had not been investigated in randomized controlled studies.

### Case 1

Case 1 is a 27-year-old woman with a major depressive episode in the context of bipolar disorder (BD) type I. The depressive episode was of moderate to severe severity without psychotic symptoms. She had failed several antidepressant and mood stabilizers including lithium during the current depressive episode. She had an acute course of nine RUL-UB pulse width treatments over 1 month and eight continuation RUL-UB pulse width treatments over 3 months. During ECT treatment, she was not prescribed any psychotropic medications. She had a greater than 50% reduction in QIDS scores at the end of her ECT course (23 to 10). She wished to stop the C-ECT course due to concerns over the disruption in her daily schedule and cognitive adverse effects. She began a course of rTMS within 1 week of her last ECT treatment and had a total 40 sessions over 6 months twice per week, that consisted of 600 pulses of LFR prefrontal rTMS at 41% of the MSO and 3000 pulses of HFL prefrontal rTMS at 43% MSO. During rTMS treatment she was prescribed trazodone for sleep, PRN lorazepam for anxiety, and zopiclone PRN. When she began rTMS (i.e., at the end of ECT) her QIDS-SR score was 13 and dropped to 10 at the end of her rTMS course (at several time points during the rTMS course her QIDS-SR score was in the remission range).

### Case 2

Case 2 is a 47-year-old woman with a major depressive episode in the context of BD type II. The depressive episode was of moderate severity and she had failed two adequate trials of an antidepressants with augmentation. She had an acute course of 18 RUL-UB pulse width treatments over 2 months and 31 continuation and maintenance RUL-UB pulse width treatments over 9 months. During ECT treatment, she was prescribed venlafaxine, nortriptyline, olanzapine, aripiprazole, lithium, and zopiclone. The venlafaxine and olanzapine were discontinued during the acute ECT course and the nortriptyline dose was optimized. She had a greater than 50% reduction in QIDS-SR scores at the end of her ECT course (13 to 3). At the end of the acute ECT course her QIDS-SR score were in the remission range. She began to notice cognitive impairments during the maintenance ECT course and wished to discontinue the treatment. Due to her history of recurrent episodes rTMS was considered. She received a total 58 sessions on a once weekly basis over 13 months that consisted of 600 pulses of LFR rTMS at 54% MSO and 3000 pulses of HFL rTMS at 46% MSO. During rTMS treatment, nortriptyline, lithium, aripiprazole, and zopiclone were prescribed with no dose changes. When she began rTMS (after stopping her maintenance ECT course) her QIDS-SR score was 2 and was maintained at 3 at the last observation.

### Case 3

Case 3 is a 58-year-old woman with a depressive episode in the context of a major depressive disorder (MDD). The depressive episode was of moderate severity. She had failed two antidepressant of adequate dose and duration. She had an acute course of 10 RUL-UB pulse width treatments. During ECT treatment she was prescribed fluoxetine. Despite achieving remission (QIDS-SR score of 3) with her acute course of ECT she did not like the anesthetic side effects and declined a course of C-ECT, she also declined the addition of lithium due to concern over side effects. Given her history of recurrent episodes a course of rTMS was offered. She received 66 sessions of rTMS twice weekly over 11 months. She received 600 pulses of LFR rTMS at 43% MSO and 3000 pulses of HFL rTMS at 52% MSO. During rTMS treatment, fluoxetine was continued. Her QIDS score was 2 at the last observation.

### Case 4

Case 4 is a 51-year-old man with a major depressive episode in the context of MDD. The depressive episode was of severe severity and failed to respond to at least two antidepressants trials of adequate dose and duration. He had an acute course of six RUL-UB pulse width treatments, but due to lack of response he was then switched to bitemporal ECT for another 14 treatments. During ECT treatment, he was prescribed mirtazapine, paroxetine, clonazepam, gabapentin, and zopiclone. The gabapentin and clonazepam were discontinued during the ECT course. He had a partial response to ECT and the severity of depression was reduced to the moderate range (QIDS-SR went from 18 to 13). Due to cognitive adverse effects he was offered a course of rTMS. As the patient lived a far distance from the hospital he was only able to attend on a once weekly basis. He received 52 rTMS treatments once weekly over 7 months that consisted of 600 pulses of LFR rTMS at 57% MSO and 3000 pulses of HFL rTMS at 57% MSO. During rTMS treatment, he took mirtazapine, paroxetine, and zopiclone. When he began rTMS (i.e., at the end of ECT) his QIDS score was 13 and dropped to 12 at the last observation, during periods of his rTMS course his QIDS score was as low as 7. Subjectively, the patient reported significant improvement in his symptoms during the rTMS course.

### Case 5

Case 5 is a 51-year-old man with a major depressive episode in the context of MDD. The depressive episode was of severe severity and he failed two antidepressants of adequate dose and duration. He received an acute course of 11 RUL-UB pulse width treatments and 15 continuation and maintenance RUL-UB pulse width treatments over 7 months. During ECT treatment, he was prescribed venlafaxine and nortriptyline. He had a partial response to the acute ECT course and the severity of the depression was reduced to the moderate range (QIDS score of 11). Due to cognitive adverse effects he was offered a course of rTMS. He received a total 65 rTMS treatments, twice per week, over 10 months that consisted of 600 pulses of LFR rTMS at 54% MSO and 3000 pulses of HFL rTMS at 57% MSO twice a week for 5 months. Due to ongoing partial response (QIDS score of 12) he was switched to 6000 pulses of HFL rTMS at 56% twice a week over the next 5 months. At periods during the 6000 pulse HFL rTMS course his QIDS score was as low as 8. At the last observation period his QIDS score remained in the moderate range at 11. The reduction of his depression scores into the moderate range was greater than the improvement he had with medication and led to the continuation of rTMS despite the partial response.

### Case 6

Case 6 is a 56-year-old woman with a major depressive episode in the context of MDD. The depressive episode was severe with psychotic features. She had an acute course of 13 RUL-UB pulse width treatments and 10 continuation RUL-UB pulse width treatments over 2 months. At the beginning of her ECT treatment, she was prescribed mirtazapine, desvenlafaxine, risperidone, and clonazepam. The venlafaxine was switched to nortriptyline and the clonazepam was discontinued during the ECT course. She had a good response to the acute course of ECT and her QIDS-SR score were in the remission range. Despite achieving remission (QIDS-SR score of 2) with ECT she had difficulty tolerating the side effects of the anesthesia, the disruption in her daily life, and wished to discontinue the C-ECT. Due to her history of a severe episode with psychotic features and her history of recurrent episode she was offered a course of rTMS. She received a total of 44 rTMS treatments, twice per week, over 6 months that consisted of 600 pulses of LFR rTMS at 82% MSO and 3000 pulses of HFL rTMS at 72% MSO. During rTMS treatment, she was prescribed mirtazapine, nortriptyline, and risperidone. At the last observation her QIDS-SR score was a 9. Despite experiencing a mild worsening in her depressive symptoms she continued to be significantly improved from her index episode.

## Summary

**Figure 1:**
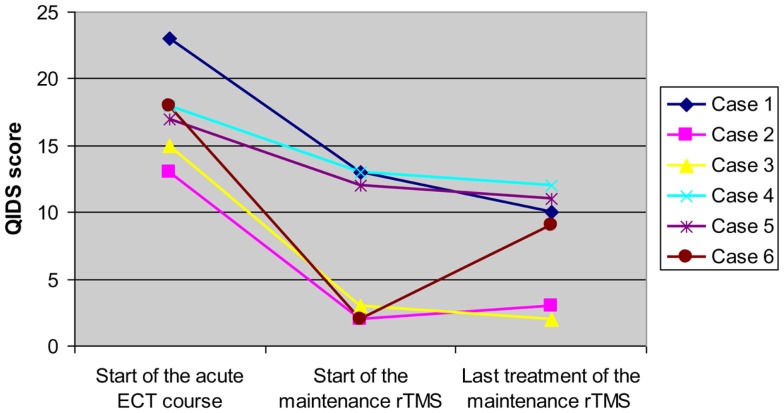
**Longitudinal changes in QIDS score of each subject over acute ECT course and maintenance rTMS treatment for refractory depression**.

Figure 1 shows the changes in QIDS-SR score throughout the treatment courses in each subject. The mean score of 8 (mild depression) was maintained through the rTMS treatment. The mean score decreased from severe to mild and was maintained in this range throughout the course of treatment. Individually, each patient achieved a significant decrease in their QIDS-SR score between the acute ECT and the rTMS courses. This decrease in QIDS-SR score translates to a change in symptom severity category. Two patients decreased by three severity categories, two decreased by two categories, and two decreased by one category. Five of the six patients maintained the response level that they had achieved during their acute and C-ECT courses. One patient (Case 6) had an increase in QIDS score from remission to the mild depression range during the rTMS treatment course. This increase in symptom level was not considered a relapse for the patient. The duration of response or partial response ranged from 6 to 13 months at the time of last observation. All patients tolerated the rTMS very well without any serious adverse effects. Two patients reported mild headache and scalp discomfort during the first several treatments but these side effects subsided quickly.

## Discussion

In this case series we present data for the effectiveness of rTMS to maintain response after achieving remission and/or response following a course of ECT in refractory depression. There is relatively little data that has investigated maintenance rTMS treatment to prevent relapse in TRD patients who have responded well to an acute course of ECT (36). This case series is in keeping with another recent case series (37). In that case series, the authors found that rTMS primarily administered as HFL, at a ratio of two rTMS to one ECT session, was effective at maintaining response in four of the six patients. Two of the six patients were switched to sequential bilateral rTMS and total pulses were increased to 6600 pulses in another two cases. Taken together, the current case series and the series presented by Cristancho et al. suggest that rTMS delivered more frequently than C-ECT may be a promising strategy to maintain response in patients who cannot tolerate or decline C-ECT and/or maintenance ECT.

The maintenance of response or partial response from the end of the ECT course ranged from 6 to 13 months at the last observation period during the rTMS course. Only one patient had a partial worsening of symptoms. The maintenance of the response for the patients in this case series was clinically significant in the context of the chronicity of their illnesses.

These results suggest that once ECT is effective for TRD, rTMS preferably at a frequency of twice per week can maintain the response achieved by ECT. All patients tolerated the treatment well. The rTMS delivered in this series was felt to be less invasive and more tolerable to the patients. The alternative evidence-based options to maintain response within the first 6 months after response from an acute ECT course can pose tolerability issues and patients often decline the two options. The adverse effect profile and tolerability of rTMS may be more appealing to patients. However, the twice weekly treatment schedule may be overly onerous to some.

There are several limitations to this case series. First, clinician-rated measures of clinical symptom severity were not collected. There are limitations to self-report measures that are not corroborated by a clinician-rated measure (38, 39). Second, longitudinal changes in cognitive function in the patients after remission with ECT course were not evaluated. As the cause for discontinuing ECT in several cases was cognitive adverse effects an assessment of cognitive function and recovery during the rTMS course would be an important measure to include in a prospective study. Third, rTMS was started after patients had already had some C-ECT; it is possible that the maintenance of response, during rTMS was affected by the previous C-ECT course. In addition, we were not able to control for the confounding effects of medication, because the type and dose of medications were not constant in these patients. However, there were no medication switches during the rTMS course. Finally, the case series is limited by its retrospective nature and the lack of a control group.

These limitations notwithstanding, this case series suggests that maintenance rTMS may be another potential treatment to maintain response after a successful course of ECT. Controlled studies comparing rTMS to current evidenced-based relapse prevention strategies such as C-ECT or C-Pharm are warranted. Such studies should include comprehensive measures of cognition.

## Conflict of Interest Statement

The authors declare that the research was conducted in the absence of any commercial or financial relationships that could be construed as a potential conflict of interest.
